# Variations in preservation of exceptional fossils within concretions

**DOI:** 10.1186/s13358-023-00284-4

**Published:** 2023-09-14

**Authors:** Farid Saleh, Thomas Clements, Vincent Perrier, Allison C. Daley, Jonathan B. Antcliffe

**Affiliations:** 1 Institute of Earth Sciences (ISTE), University of Lausanne, Geopolis, CH-1015 Lausanne, Switzerland; 2 GeoZentrum Nordbayern, Friedrich-Alexander Universität Erlangen-Nürnberg, Loewenichstrasse 28, 91054 Erlangen, Germany; 3 Université de Lyon, UCBL, ENSL, CNRS, UMR 5276 LGL-TPE, 69622 Villeurbanne, France

**Keywords:** Concretions, Exceptional fossils, Silurian, Carboniferous, Konservat-Lagerstätte

## Abstract

Concretions are an interesting mode of preservation that can occasionally yield fossils with soft tissues. To properly interpret these fossils, an understanding of their fossilization is required. Probabilistic models are useful tools to identify variations between different Konservat-Lagerstätten that are separated spatially and temporally. However, the application of probabilistic modeling has been limited to Early Paleozoic Konservat-Lagerstätten preserved in shales. In this paper, the patterns of preservation of three concretionary Konservat-Lagerstätten—the Carboniferous Mazon Creek (USA) and Montceau-les-Mines (France), and the Silurian Herefordshire Lagerstätte (UK)—are analyzed using a statistical approach. It is demonstrated that the degree of biotic involvement, i.e., the degree to which a carcass dictates its own preservation, is connected to internal organ conditional probabilities—the probabilities of finding an internal organ associated with another structure such as biomineralized, sclerotized, cuticularized, or cellular body walls. In concretions that are externally forced with little biological mediation (e.g., Herefordshire), all internal organ conditional probabilities are uniform. As biological mediation in concretion formation becomes more pronounced, heterogeneities in conditional probabilities are introduced (e.g., Montceau-les-Mines and Mazon Creek). The three concretionary sites were also compared with previously investigated Konservat-Lagerstätten preserving fossils in shales to demonstrate how the developed probability framework aids in understanding the broad-scale functioning of preservation in Konservat-Lagerstätten.

**Supplementary Information:**

The online version contains supplementary material available at 10.1186/s13358-023-00284-4.

## Introduction

The experience of walking in nature and cracking open concretions in search of fossils captures the joy of paleontology. Every concretion split open has the potential to reveal a, typically exceptionally preserved, fossil. People have been walking, searching, spotting oddly shaped rocks, cracking them open, and eagerly anticipating the fossil treasures within for centuries. In fact, Charles Darwin (1846), during his voyage on HMS Beagle (1832–1836), examined fossiliferous concretions found in South America around the Chilean Cordillera and the Pampas:

“*Dr. Carpenter has kindly examined under the microscope, sliced and polished specimens of these concretions, and of the solid marl-rock, collected in various places between the Colorado and Santa Fé Bajada. In the greater number, Dr. Carpenter finds that the whole substance presents a tolerably uniform amorphous character… In some, Dr. Carpenter can perceive distinct traces of shells, corals, Polythalamia, and rarely of spongoid bodies. Dr. Carpenter informs me that it is well known that chemical precipitation throws down carbonate of lime in the opaque amorphous state… I can hardly doubt that the amorphous carbonate of lime in them has resulted from the attrition and decay of the larger fragments of shell… the amorphous matter in the marly rocks of the Pampas, has likewise thus originated … [though] … it would be hazardous to conjecture.*” (Darwin, 1846, page 76).

**Fig. 1:**
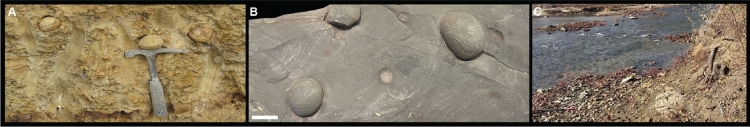
Unopened concretions from the Herefordshire Lagerstätte (**A**) and Montceau-les-Mines (**B**) in addition to the concretionary level of the Mazon Creek under water (**C**). Scale bar represents 2 cm in **B**

**Fig. 2:**
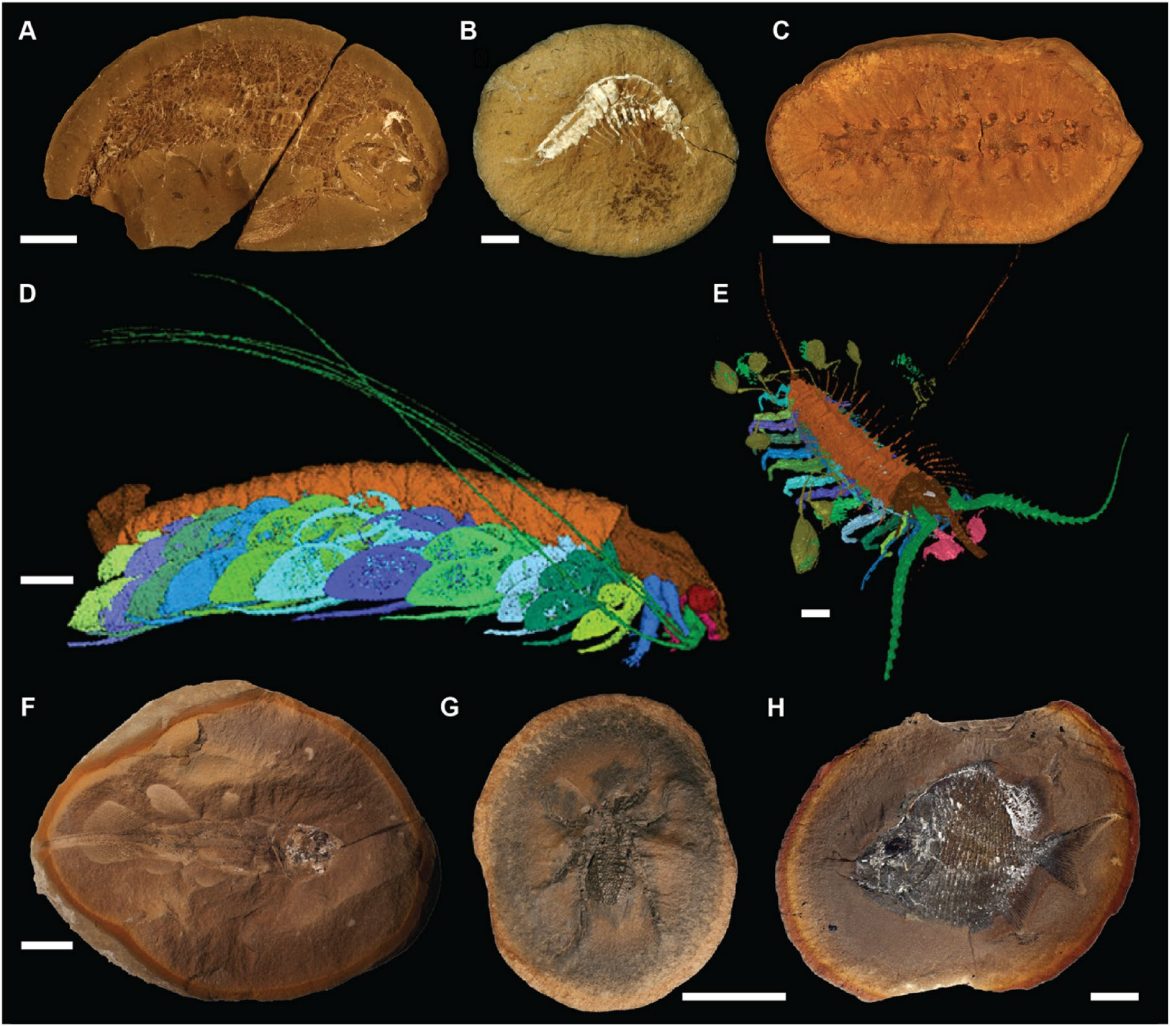
Fossils from the Montceau-les-Mines, Herefordshire, and the Mazon Creek Lagerstätten. A–C Montceau-les-Mines: **A** Undescribed actinopterygian MNHN.SOT 001568a, **B** *Palaeocaris secretanae* (Schram, 1984) MNHN.SOT 024746a, **C** *Palaeocampa anthrax* (Meek & Worthen, 1865) MNHN.SOT 003657a. **D**, **E** Herefordshire: **D** *Cascolus ravitis* (Siveter et al., 2017) OUMNH C.29698, **E** *Aquilonifer spinosus* (Briggs et al., 2016) OUMNH C.29695. **F**–**H** Mazon Creek (Clements et al., 2019): **F** *Rhabdoderma* sp. ROM56774, (**G**) *Aphantomartus pustulatus* BMRP2014MCP850, **H** *Platysomus circularis* FMNHPF7333. Scale bars represent 1 cm in **A**, **F**, **G,** and **H**; 5 mm in **B** and **C**; 500 µm in **D** and **E**; OUMNH numbers are registered at Oxford University Museum of Natural History; MNHN: Museum National d’Histoire Naturelle d’Autun; ROM: Royal Ontario Museum; FMNHPF: Field Museum of Natural History; BMRP: Burpee Museum of Natural History

It has long been recognized that concretions are unusual in their preservation as they are quite different from typical fossils consisting of “shells and bones.” Typically, fossil-bearing concretions are formed of carbonates (although they can also be formed from phosphate or silicate) that precipitate around a locus, such as an animal carcass, within lithifying sediment (McCoy et al., 2015). They can also form without a locus due to pronounced chemical gradients. The source of the concretionary material and its connection to the degradation of the fossils trapped within are as challenging to comprehend today as they were to Charles Darwin in 1846 and some questions remain about concretion preservation: what exactly controls the onset of concretion growth, what controls the growth speed of concretions, why some are more fossiliferous than others, and how can they be examined in the context of other exceptional modes of preservation. To answer these questions, the preservation of three Konservat-Lagerstätten preserving ancient biotas within concretions (see Figs. 1, 2) was investigated. These sites are the Silurian Herefordshire Lagerstätte (Welsh Basin, UK), and the Carboniferous Mazon Creek (Illinois, USA) and Montceau-les-Mines (Bourgogne-Franche-Comté, France). These sites allow investigation of concretionary preservation from a range of paleoenvironmental conditions.

The Carboniferous Mazon Creek Lagerstätte is a geographically vast (250 km^2^) deposit in which fossils are preserved within siderite (FeCO_3_) concretions (Clements et al., 2016, 2017, 2019; Richardson & Johnson, 1971; Schram, 1979; Shabica, 1979). This Lagerstätte records a tidally influenced equatorial nearshore marine basin (Clements et al., 2019). Large amounts of sediments and organic matter were deposited by large river systems into the marine basin, inundating and burying organisms inhabiting the bay. The Mazon Creek Lagerstätte is famous for the incredible diversity of preserved organisms and plants (encompassing reptiles, amphibians, fish, arthropods, polychaetes, chitons, jellyfish, and even enigmatic forms such as the famous *Tullimonstrum*), the exceptional (often three dimensional) soft tissue preservation with little disarticulation (Fig. 2), and the sheer quantity of fossils that have been extracted from the site since the 1950s—some estimate millions of fossiliferous concretions (Clements et al., 2019). These factors contribute to the notion that the Mazon Creek Lagerstätte is one of the best fossil deposits in the world providing unique data on terrestrial and marine ecosystems during the Carboniferous (see Clements et al., 2019).

The contemporaneous Montceau-les-Mines Lagerstätte (Charbonnier & Chabard, 2014; Heyler & Poplin, 1988; Rolfe et al., 1982) is situated in a 40 km long intra-mountainous basin representing fluvial, deltaic, and lacustrine equatorial environments. During tropical storms, terrestrial fauna and flora were washed into the basin and rapidly buried together with aquatic organisms (Fig. 2). From 1970 to 1982, the coal exploitation in the Montceau-les-Mines basin led to the discovery and collection, by the amateur paleontologist Daniel Sotty and his team, of more than 120,000 fossiliferous concretions (Sotty, 1980). These fossils (e.g., reptiles, amphibians, fishes, arthropods, onychophorans, annelids), preserved within sideritic concretions, are typically preserved with 3D soft tissues (e.g., digestive tracts, skin), or extremely fragile cuticular features such as appendages and setae. These fossils provide a unique window into equatorial freshwater and terrestrial late Carboniferous ecosystems (Perrier & Charbonnier, 2014).

The third Lagerstätte is the mid-Silurian Herefordshire Lagerstätte (Welsh Basin, UK) which was deposited at tropical/subtropical paleolatitudes (Briggs et al., 1996). This Lagerstätte represents an outer shelf/shelf slope marine environment that was inundated by volcaniclastic sediment (Orr et al., 2000). The Herefordshire Lagerstätte likely represents the distal eruption products of Silurian volcanoes related to the final closure of the Iapetus Ocean (Holland, 1988). As such, the fossils are preserved as calcitic void infills in early diagenetic carbonate concretions within a volcaniclastic (bentonite) horizon. Yielding a wide range of invertebrates (e.g., arthropods, sponges, brachiopods, mollusks, echinoderms; Siveter et al., 2020) (Fig. 2), this exceptional Lagerstätte provides a range of currently unique fossil organisms, for example, the only known gastropod with soft parts in the fossil record (Sutton et al., 2006), pycnogonids (sea spider; Siveter et al., 2004), and the only fossil parasitic pentastomid (tongue worm) preserved with its host (Siveter et al., 2015).

These fossil sites, as with other Konservat-Lagerstätten (Brasier et al., 2011; Seilacher, 1970), are vital for reconstructing ancient ecosystems (e.g., Barling et al., 2015; Charbonnier et al., 2014; Fu et al., 2019; Nanglu et al., 2020; Piñeiro et al., 2012; Saleh et al., 2021a; Südkamp, 2007; von Bitter et al., 2007). However, even in fossil sites where labile morphological structures are commonly preserved, we must be aware that the fossil record is subject to information loss due to the taphonomic processes that occur prior to geological stabilization of soft tissues, e.g., transport and decay (see Purnell et al., 2018). The degree to which these processes operate differs between fossil sites due to the large number of variables that govern preservation, especially that of soft tissues. Recent taphonomic approaches (e.g., Reeves & Sansom, 2023), including probability-based models (e.g., Saleh et al., 2020) based on presence–absence of tissue types, provide novel tools to identify patterns of fossil preservation and allow statistical comparison between fossil sites—even those that were not deposited contemporaneously or that do not have the same mode of preservation. This methodology has been used to compare sites that exhibit Burgess Shale-type soft tissue preservation in shales in the Cambrian (Burgess Shale, Canada; Chengjiang, China; Spence Shale, USA) and Ordovician (Fezouata Biota, Morocco) (Saleh et al., 2020, 2021b, 2022a; Whitaker et al., 2022), but has never been applied to any site preserving exceptional fossils within concretions.

It is important to note that this work does not seek to rank exceptionally preserved biotas from the best to the worst preserved. Probabilistic models serve to investigate preservation variations between sites. By identifying the probability of tissue occurrences and co-occurrences, this study can help identify taphonomic processes that occurred during preservation in Konservat-Lagerstätten. This allows us to model processes that operate during fossilization at these sites and compare them to other exceptionally preserved fossil sites such as Burgess Shale-type (BST) biotas, which are already well constrained by probabilistic models.

## Materials and methods

Lists of eumetazoan genera preserved in the Mazon Creek, Montceau-les-Mines, and Herefordshire Lagerstätten were compiled from figures of published specimens (e.g., Clements et al., 2019; Perrier & Charbonnier, 2014; Shabica & Hay, 1997; Siveter et al., 2020), as well as from the collections of the Autun Museum of Natural History (France) for the Montceau-les-Mines data. The raw data, including generic lists and identified morphological characters, are provided in the supplementary data file. For more enigmatic taxa where there has been some debate about their affinity, we used the most recently published phylum placement. It is noted that the Montceau-les-Mines database does not account for the numerous fishes and juvenile insects discovered because these were preserved in shales and not within concretions.

To investigate the variation in preservation, biological tissues were divided into five categories based on their generalized resistance to the decay process as per Saleh et al., (2020): A, biominerals; B, sclerotized cuticles; C, non-sclerotized cuticles; D, external epidermis in direct contact with the surrounding environment; and E, internal organs. The five categories of tissue types describe the original anatomical composition of organisms rather than the result of a specific taphonomic pathway. For example, a pyritized digestive system would be classified under Category E, which encompasses internal organs, rather than Category A, which is for biomineralized structures. Below are general examples for each category in the animal kingdom.

Category A is for biomineralized structures, regardless of the nature or degree of mineralization. This category includes structures such as trilobite and echinoderm exoskeletal elements, as well as the shells of groups such as hyoliths, mollusks, and brachiopods.

Category B is for sclerotized structures that are hard or plate like. This category includes the rhabdosome/tubarium of graptolites, the chaetae of annelids, the carapaces of many bivalved arthropods, and some other body parts of arthropods (e.g., antennae and ventral appendages of trilobites).

Category C is for non-sclerotized cuticularized bodies formed of polysaccharides. This category includes the body walls of animals such as annelids and priapulids, in addition to parts of non-biomineralized arthropods (e.g., arthrodial membranes).

Category D describes body parts that are composed solely of cells without a layer of polysaccharides and without any mineralogical enrichment, and are in direct contact with the surrounding environment. This category includes the body envelope of animal groups such as chordates and jellyfish, in addition to the tentacles of cnidarians, brachiopods, and hyoliths.

Category E is for internal organs and systems, such as the digestive, respiratory, nervous, and reproductive systems, as well as the musculature of any animal group.

To determine the occurrences of tissues for each genus, the best-preserved specimen of the genus or a combination of specimens is taken into account. For instance, if there are 99 fragmentary samples and only one complete specimen for a certain genus, the complete specimen is used to fill in the database. If another genus has two specimens, one shows a biomineralized structure and a gut, while the other preserves a non-sclerotized cuticle, this genus is considered to preserve biominerals, non-sclerotized cuticles, and internal organs.

The total number of genera having just one character (“1 s”; i.e., A, or B, or C, or D, or E) was plotted against the number of genera that have two (“2 s”; e.g., AB), three (“3 s”; e.g., ABC), or four (“4 s”; e.g., ABCE) characters. The proportion of each of the different possible tissue-type combinations was calculated [e.g., P(A ∩ B); P(A ∩ B ∩ E)].

To examine how exceptional preservation differs between the three sites, mainly the preservation of internal organs (E) was considered. This category was chosen because it must have been present during the life of all examined eumetazoans, whereas this is not true for all the other categories. For example, it is possible to have an animal with no biominerals. The association of internal organs (E) with other structures was investigated for all tissue-type categories by examining conditional probabilities. For instance, P(E|A) is the probability of finding an internal organ given that a biomineralized structure has been found. To investigate whether the differences in conditional probabilities between sites are significant, a binomial model was used. For an overview of the methodology, see Saleh et al., (2021b).

## Results and discussion

### Preservation patterns in concretions

**Fig. 3:**
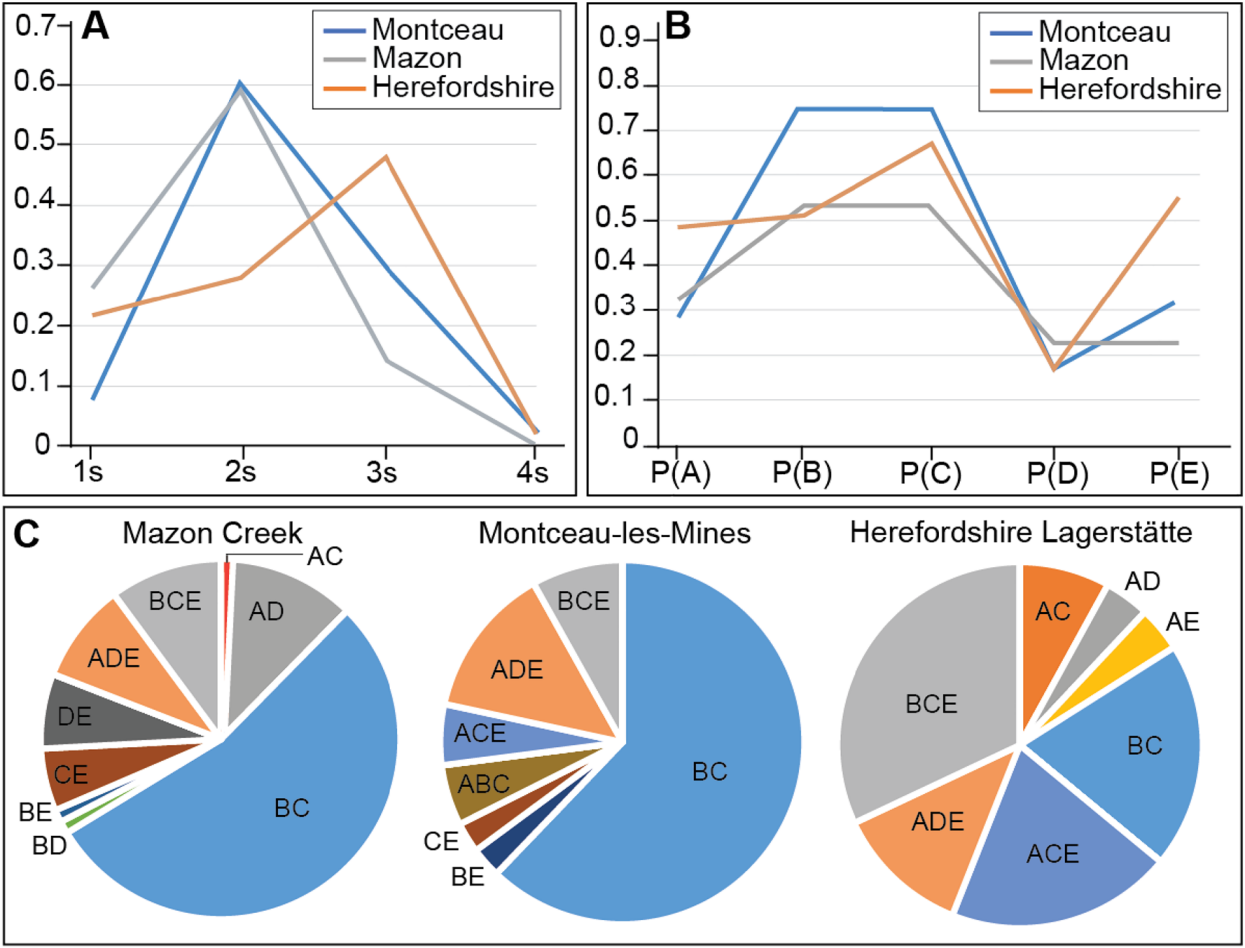
Preservation variations between the Herefordshire, Montceau-les-Mines, and Mazon Creek Lagerstätten. **A** Taxa preserving one type of tissue (1 s); two, three, and four types of tissues (2 s, 3 s, and 4 s, respectively). **B** The proportion of each tissue category [i.e., biominerals P(A); sclerite P(B); cuticle P(C); cellular sheets in direct contact with the surrounding environment P(D); and internal organs P(E)]. **C** Proportions of tissue associations

The summary data from the three investigated sites are given in Table 1. The Herefordshire Lagerstätte shows a higher number of genera preserving a combination of three types of structures “3 s” (in proportion to 1 s, 2 s, and 4 s) when compared to Montceau-les-Mines and the Mazon Creek—in these two latter sites, genera tend to preserve mostly a combination of two types of structures (“2 s”; Fig. 3A). Herefordshire also preserves a higher proportion of internal organs P(E) when compared to Montceau-les-Mines and the Mazon Creek (Fig. 3B). This pattern of P(E) is also observed when taking a certain group of animals. For instance, arthropods preserve more internal organs in the Herefordshire [P(E) = 0.55] than in the Montceau-les-Mines [P(E) = 0.13] and the Mazon Creek [P(E) = 0.05]. Overall, Montceau-les-Mines and the Mazon Creek are more similar to each other in terms of tissue associations (albeit with some minor differences) than to the Herefordshire Lagerstätte (Fig. 3C).

**Table 1:** Summary statistics for Montceau-les-Mines, Mazon Creek, and Herefordshire Lagerstätten

	Mazon Creek	Montceau-les-Mines	Herefordshire
1 s	34	3	7
2 s	72	25	9
3 s	17	12	16
4 s	0	1	1
5 s	0	0	0
A	20	2	3
B	8	1	2
C	5	0	1
D	1	0	1
E	0	0	0
AB	0	0	0
AC	1	0	2
AD	11	0	1
AE	0	0	1
BC	47	23	5
BD	1	0	0
BE	1	1	0
CE	5	1	0
DE	6	0	0
ABC	0	2	0
ABD	0	0	0
ABE	0	0	0
ACE	0	2	4
ADE	8	5	3
BCE	9	3	8
BDE	0	0	0
P(A)	0.325203252	0.292682927	0.484848485
P(B)	0.536585366	0.756097561	0.515151515
P(C)	0.544715447	0.756097561	0.666666667
P(D)	0.219512195	0.146341463	0.151515152
P(E)	0.235772358	0.317073171	0.545454545
P(E|A)	0.2	0.666666667	0.625
P(E|B)	0.151515152	0.161290323	0.5882353
P(E|C)	0.208955224	0.193548387	0.636363636
P(E|D)	0.518518519	1	0.6

A = biominerals, B = sclerites, C = cuticle, D = cellular body walls, and E = internal organs. Whole integers indicate numbers of genera belonging to each category (e.g., A, AB, ABE). 1 s, 2 s, 3 s, and 4 s indicate the number of taxa having one character (A, or B, or C, or D, or E), two characters (e.g., AB), three characters (e.g., ABC), and four characters (e.g., ABCE), respectively. The proportions of each type of tissue in all categories (e.g., P(A), P(C)) are calculated. Probabilities of finding internal tissue in association with other structures (e.g., P(E|A)) are calculated as well

One plausible explanation for these differences may be the application, and limitation, of 3D imaging methods used to investigate fossil anatomy in these sites. When analyzed using non-destructive X-ray computed tomography, taxa from Montceau-les-Mines, particularly arthropods, can yield exquisite 3D data allowing detailed morphological reconstructions (Garwood et al., 2011, 2012, 2016; Lheritier et al., 2023). In contrast, Mazon Creek fossils are notoriously difficult for CT scan, as the fossils often have low-density contrast to the matrix (McCoy, 2015). This is also the case for Herefordshire fossils, which have a low-density contrast between the calcite infills of fossil organisms and the surrounding carbonate matrix (Siveter et al., 2020). To counter this issue, investigations of the Herefordshire fossil fauna use the destructive physical–optical tomography “serial grinding” technique which has allowed anatomies to be reconstructed at high resolution (e.g., Briggs et al., 2012; Sutton et al., 2001). Due to its destructive and time-consuming nature, the serial grinding method has not been used to investigate concretions from Montceau-les-Mines or the Mazon Creek. Despite the use of 3D imaging methods allowing greater anatomical resolution of fossils from Herefordshire and Montceau-les-Mines, this is somewhat negated by the sheer amount of fossil material that has been found from the Mazon Creek, which allowed detailed anatomical descriptions of many fossil species.

**Fig. 4:**
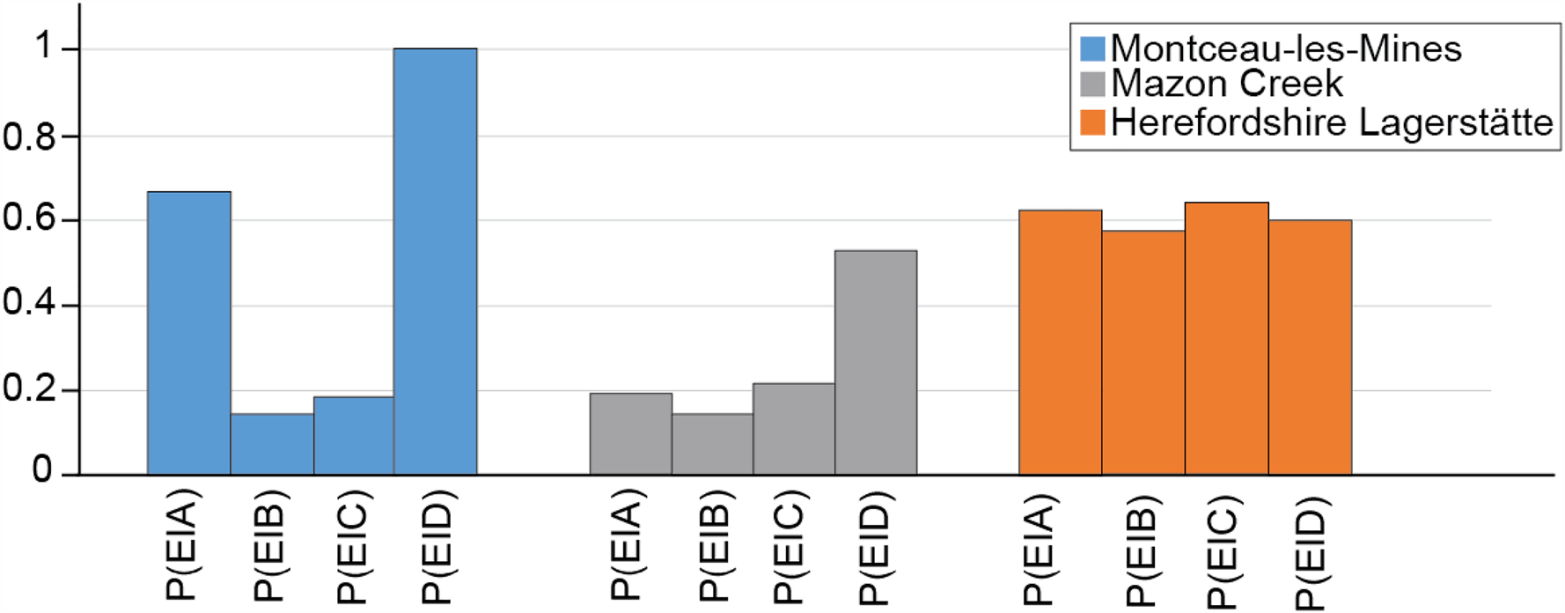
Conditional probabilities showing the association of internal organs with biominerals P(E|A); sclerite P(E|B); cuticle P(E|C); and cellular sheets in direct contact with the surrounding environment P(E|D)

The differences between the three investigated sites could also result from different taphonomic pathways. Although all are concretionary fossil sites, there are differences in their mode of preservation. The Herefordshire Lagerstätte preserves fossils within volcanoclastic concretions, whereas the other two sites are siderite concretion-bearing Konservat-Lagerstätten. Differences between Montceau-les-Mines and the Herefordshire Lagerstätte, particularly in P(E), can be linked to the fact that many fossils from Montceau-les-Mines have considerably decayed or emptied as a preparation for latex casting and thus consist of voids (e.g., Garwood et al., 2012), while this is not the case in the Herefordshire Lagerstätte. Moreover, even though Montceau-les-Mines and the Mazon Creek are relatively contemporaneous and preserve soft tissue fossils within siderite concretions, there are differences in their respective preservational paleoenvironments (see Introduction). Thus, despite concretion formation being preliminarily controlled by the decay of organic matter, the differing environmental factors at these sites are likely to have impacted their formation. To investigate the preservation differences in details between the three sites, conditional probabilities are required (Fig. 4). For instance, P(E|D), or the probability of finding internal organs (E) considering that external epidermis (D) has been identified, is the highest in Montceau-les-Mines (Fig. 4). For the conditionals of sclerites (B), and cuticles (C), with internal organs (E) [i.e., P(E|B) or the probability of finding internal organs given that the genus has preserved sclerotized parts, and P(E|C) or the probability of finding internal organs given that the genus has preserved cuticularized parts], Herefordshire shows the highest probabilities (Fig. 4). Regarding the association of biominerals (A) and internal organs (E), Mazon Creek has the lowest P(E|A) (Fig. 4). These differences between sites are statistically significant (Table 2).

**Table 2:** *p*-values for trying to reproduce patterns of preservation in one Lagerstätte based on the distribution of characters in the two other Lagerstätten for the four conditional probabilities

		Using data from	
		Montceau-les-Mines	Herefordshire
**Replicating values of Mazon Creek**	P(E|A)	0.00410	< 0.00001
	P(E|B)	0.18924	< 0.00001
	P(E|C)	0.57162	< 0.00001
	P(E|D)	< 0.00001	0.20100
		**Mazon Creek**	**Herefordshire**
**Replicating values of Montceau-les-Mines**	P(E|A)	< 0.00001	0.19543
	P(E|B)	0.62438	< 0.00001
	P(E|C)	0.37326	< 0.00001
	P(E|D)	< 0.00001	< 0.00001
		**Mazon Creek**	**Montceau-les-Mines**
**Replicating values of Hereforshire**	P(E|A)	< 0.00001	0.49583
	P(E|B)	< 0.00001	< 0.00001
	P(E|C)	< 0.00001	< 0.00001
	P(E|D)	0.10557	0.04701

If *p* is < 0.05 this means that the conditional probabilities cannot be reproduced, and the difference is significant. If *p* is > 0.05, the conditional probabilities can be reproduced, and the difference is not significant. It is noted that P(E|A) for the Mazon Creek is significantly different from both Montceau-les-Mines and Herefordshire Lagerstätte. P(E|B) and P(E|C) are significantly different between the Herefordshire Lagerstätte on one hand and Montceau-les-Mines and Mazon Creek on the other hand. For P(E|D), the difference is significant between Montceau-les-Mines and the two other Lagerstätten

### Taphonomic processes responsible for varying concretion preservation

**Fig. 5:**
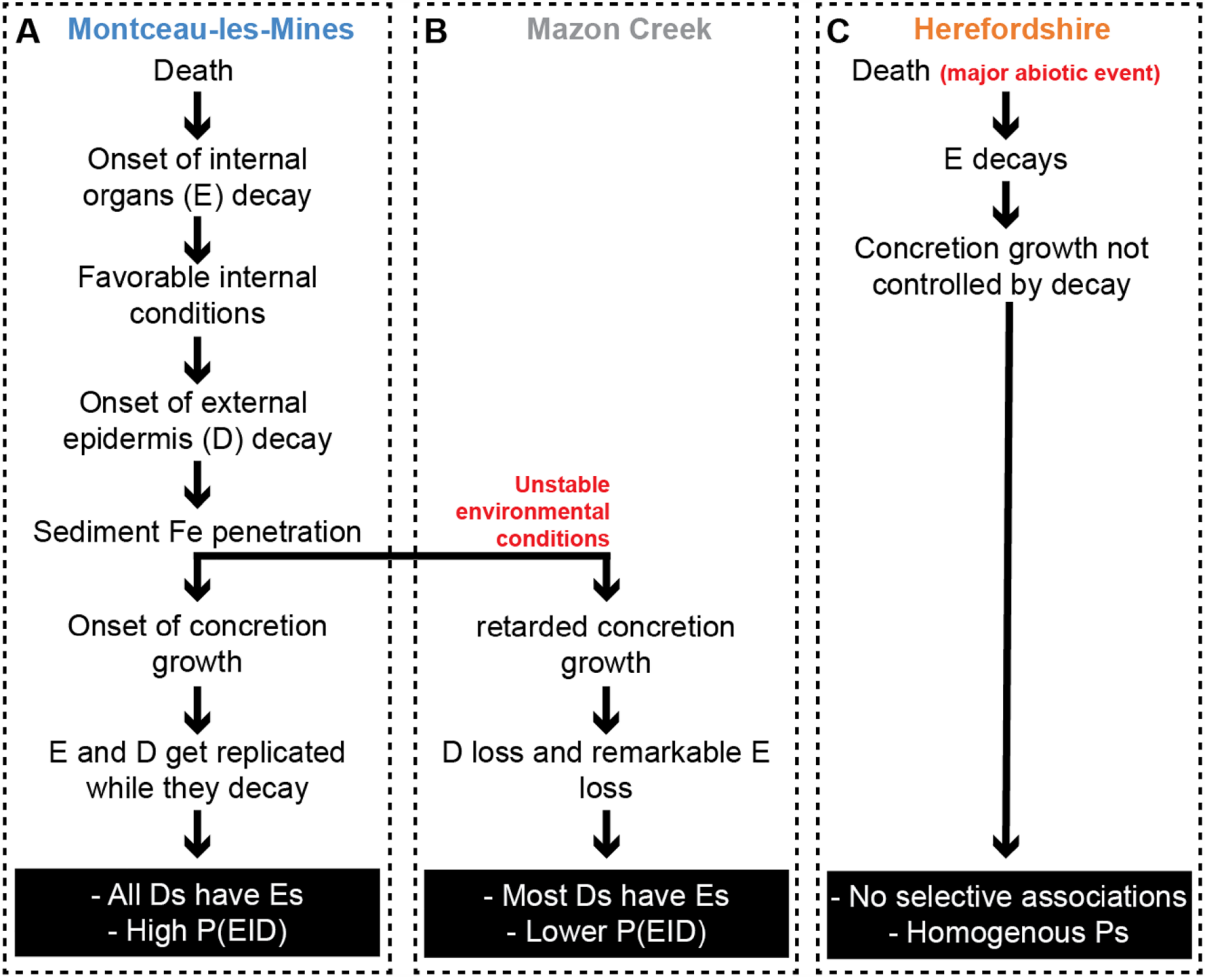
Preservational models explaining the heterogeneities between (**A**) Montceau-les-Mines, (**B**) Mazon Creek, and (**C**) Herefordshire Lagerstätten. Cellular sheets in direct contact with the surrounding environment “D”; and internal organs “E”; “P” is for probability. P(E|D) is the probability of finding internal organs (E) considering that external epidermis (D) has been identified

Following the death of an organism it starts to decay. Decay experiments on extant animals show that internal organs are typically the fastest tissues to decay in oxygenated artificial sea water (e.g., Clements et al., 2022; Murdock et al., 2014; Sansom, 2016; Sansom et al., 2010) (Fig. 5A). The decay of the organism alters the geochemical conditions (e.g., pH) within and around its decaying carcass (Clements et al., 2019, 2022; Sagemann et al., 1999). In Montceau-les-Mines and the Mazon Creek, the decaying organics created a favorable environment for siderite precipitation (Clements et al., 2019) (Fig. 5A). In both sites, the high level of iron-rich terrigenous sediment that buried organisms allowed the formation of “proto-concretions” (see Baird et al., 1986; Clements et al., 2019 and references therein). Considering that an epidermis in direct contact with the surrounding environment (D) decays faster than biomineralized, sclerotized, and cuticularized body walls (Briggs, 2003; Hancy & Antcliffe, 2020; Tegelaar et al., 1989; Fig. 5A), Fe from the sediments could potentially penetrate the body cavity of an organism with an external epidermis more expediently than is possible for an organism with a robust exoskeleton (Fig. 5A). In this scenario, if siderite precipitation happens rapidly enough, internal organs and the epidermis would have only partially decayed and become stabilized via siderite replacement, resulting in a high P(E|D) value (Fig. 5A). As biomineralized, sclerotized, and cuticularized structures are slower to decay, longer times are needed to allow the penetration of Fe from the sediment to the internal environment. By that time, internal organs continue to decay and can disappear resulting in the lower values of P(E|A), P(E|B), and P(E|C) in comparison to P(E|D) (Fig. 5A). Our data in both Montceau-Les-Mines and the Mazon Creek support this (Fig. 4).

Our data also highlight a preservation variation between the two sideritic Konservat-Lagerstätten. In Montceau-les-Mines all external epidermis (D) are associated with internal organs (E) [P(E|D) = 1]. This is not the case for the Mazon Creek, which has a much lower E|D score [P(E|D) = 0.43] (Fig. 4). We propose that, despite both sites having the same primary mode of preservation, environmental factors may have contributed to the rate of siderite formation, and this would impact the likelihood of internal organs being preserved. Montceau-les-Mines was a freshwater depositional environment (Perrier & Charbonnier, 2014), better for the formation of siderite than the Mazon Creek Lagerstätte that was deposited in a tidally influenced delta (see Clements et al., 2019), with fluctuating environmental conditions (i.e., cyclic laminations are observed within the host sediment; Baird et al., 1986). These environmental conditions are much more unstable for the precipitation of siderite (Fig. 5B), due to the higher energy levels and the presence of seawater sulfate which acts to poison siderite formation (Clements et al., 2019). It is therefore likely that siderite formation was slower at Mazon Creek, and more internal organs were lost to decay prior to concretion stabilization than in the Montceau-les-Mines scenario (Fig. 5B).

In contrast to the sideritic Konservat-Lagerstätte, concretion growth in the Herefordshire Lagerstätte does not seem to be dependent on the generation of geochemical conditions by decaying carcasses. Fossils found at this site are not thought to be the loci of concretion formation, and often appear to have been spatially incorporated in random positions within their concretions (Siveter et al., 2020). The Herefordshire Lagerstätte represents organisms rapidly smothered by a major volcanic event. This led to homogeneous conditional probabilities (Fig. 5C) with little biological mediation and therefore no differentiation of the conditional probabilities by tissue type. Some decay did occur, hence why the conditional probabilities for this site are less than one (Fig. 4).

The previously discussed data suggest three broad scenarios of concretion preservation.

**Fig. 6:**
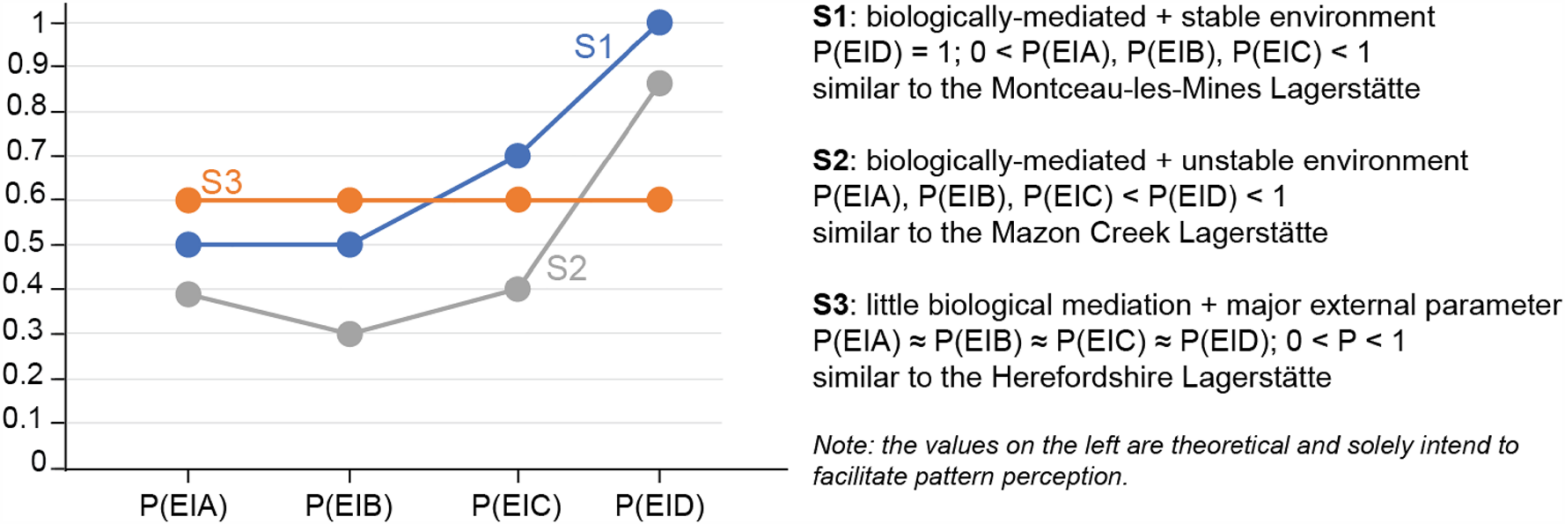
Simplified model to interpret conditional probabilities in sites preserving soft tissues within concretions. Both S1 and S2 share a biologically mediated preservation, with little external forcing, while S3 is controlled by a major external parameter and little biological mediation. S1 exhibits generally more stable environmental conditions than S2

- Scenario 1: A high association of internal organs with cellular sheets in contact with seawater [i.e., P(E|D) = 1, and higher than P(E|A), P(E|B), P(E|C)] in biologically mediated concretions under stable environmental conditions (Fig. 6; S1).
- Scenario 2: A relatively high association of internal organs with cellular sheets in contact with seawater [i.e., P(E|D) < 1 but still higher than P(E|A), P(E|B), P(E|C)] in biologically mediated concretions under unstable environmental conditions (Fig. 6; S2).
- Scenario 3: All conditional probabilities are more or less the same in the presence of a major external factor limiting the control of the carcass on its own preservation in concretions (Fig. 6; S3). The smaller the values of these probabilities are, the more degradation has occurred prior to preservation.

### Comparison with shales preserving soft tissues

**Fig. 7:**
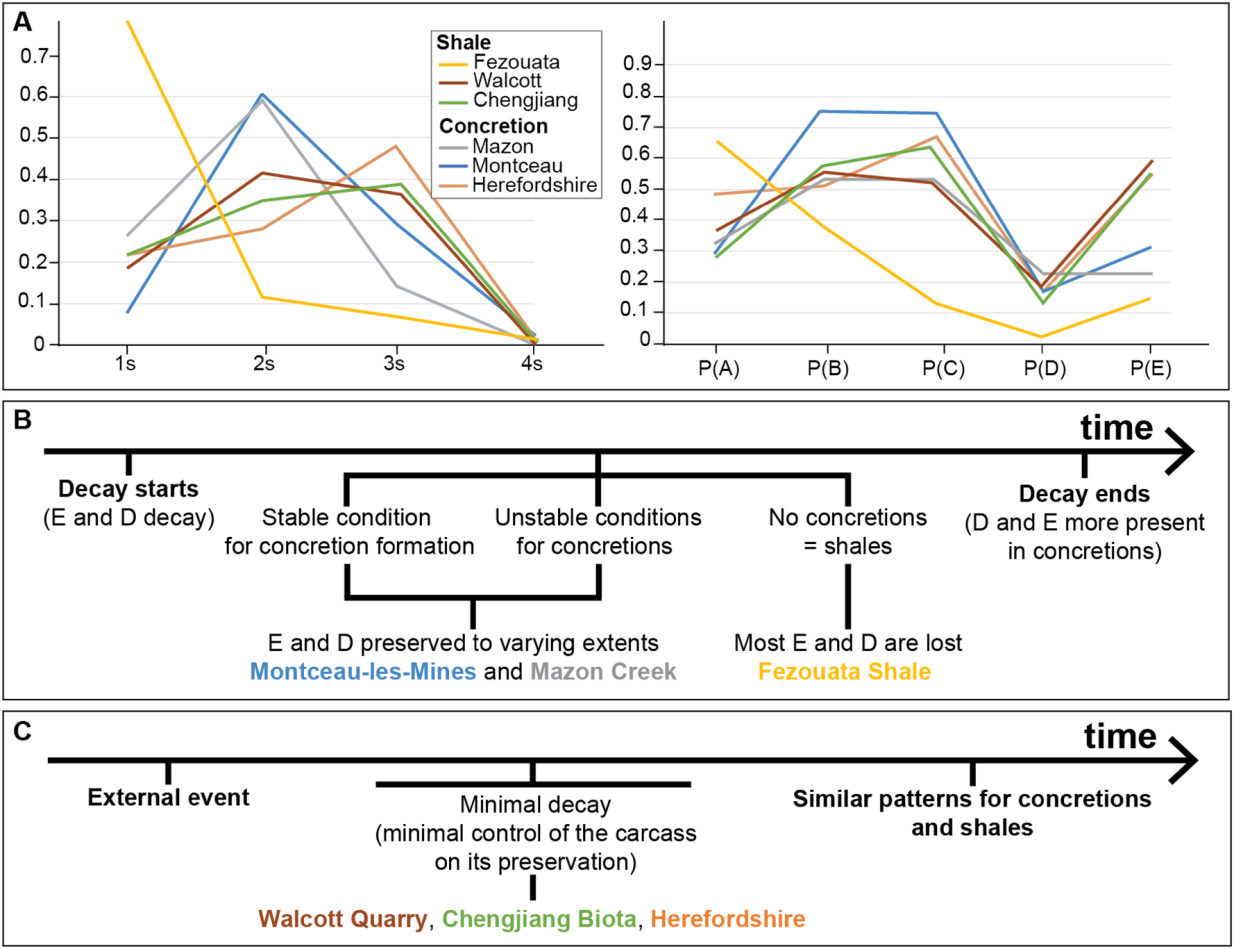
**A** Comparison between shale and concretion preservation. Taxa preserving one type of tissue (1 s); two, three, and four types of tissues (2 s, 3 s, and 4 s, respectively). The proportion of each tissue category [i.e., biominerals P(A); sclerite P(B); cuticle P(C); cellular sheets in direct contact with the surrounding environment P(D); and internal organs P(E)]. **B** Model explaining the differences between shales and concretions when carcass decay plays a major role in the fossilization process. **C** Model explaining the homogeneities between shales and concretions when decay is minimal

When compared to sites preserving soft tissues in shales, it becomes clear that the Herefordshire Lagerstätte has a similar pattern of preservational probability to both the Chengjiang Biota and the Walcott Quarry (Burgess Shale) characterized by a large proportion of 3 s and internal organs (Es) (Saleh et al., 2020; Fig. 7A). The preservation of Montceau-les-Mines and the Mazon Creek is different from all three shale sites including the Fezouata Shale (Fig. 7A). For instance, both Montceau-les-Mines and the Mazon Creek have fewer 1 s and more external cellular sheets (Ds) and internal organs (Es) in comparison to the Fezouata Shale, and more 2 s and fewer internal organs (Es) than the Walcott Quarry and the Chengjiang Biota (Fig. 7A).

In order to explain these differences, the six Konservat-Lagerstätten are divided between two groups. The preservation in the first group is not controlled by a major abiotic event (e.g., Fezouata, Montceau, and Mazon), while the preservation of the second group is dictated by major abiotic parameters such as obrution events or volcanic activity (e.g., Herefordshire, Chengjiang, and Walcott). In the absence of a major abiotic event favoring preservation, decay starts (Fig. 7B). Internal organs and the epidermis decay faster than mineralized, sclerotized, and cuticularized parts (Fig. 7B). If the surrounding environmental conditions become favorable for concretion growth (i.e., establishment of the right chemical gradients and burial by sediments after being exposed to some time on the seafloor), external cellular sheets (Ds) and internal organs (Es) will be retained, to varying extents, while they decay (i.e., preservation happens at the same time as carcass recycling because it is depending on the produced organic matter), leading to similar patterns to Montceau-les-Mines and Mazon Creek (Fig. 4; 7B). If the environmental conditions are not favorable for concretion growth (i.e., because metazoans remained unburied for a longer period of time, or the chemical conditions permissive for concretion growth were not established), external cellular sheets (Ds) and internal organs (Es) will be lost, as is the case for the Fezouata Shale (Fig. 4B; Saleh et al., 2020). In this sense, in the absence of a major controlling abiotic forcing, concretions tend to preserve considerably more external cellular sheets (Ds) and internal organs (Es), and consequently fewer 1 s than shales (Fig. 7A). It is noted that this model and the originally published database for the Fezouata Biota focuses only on fossils preserved in shales. This database did not account for the large three-dimensional carcasses of *Aegirocassis* from traditional localities near Zagora, which are preserved in biologically mediated concretions (Gaines et al., 2012), and did not consider the recently discovered locality that is dominated by concretions in Taichoute (Saleh et al., 2022b). Also it is noted that although a volcanic activity was suggested for the preservation of some animals in the Fezouata Shale, it was recently shown that  favorable elements for preservation in this site such as Fe resulted from Early Ordovician continental weathering rather than volcanism (Saleh et al., 2022c). Further investigation of whether shales and concretions differ in their preservation within the Fezouata Shale could test our hypotheses presented here.

When exceptional fossil preservation is controlled by rapid external parameters (i.e., volcanism, transport of animals and burial in an environment favoring preservation; Saleh et al., 2021b, 2022d; Siveter et al., 2020), the decay of the carcass itself plays a lesser role in controlling preservation (Fig. 7C), as discussed in the previous section for the Herefordshire and in Saleh et al. (2020) for the Chengjiang Biota and the Walcott Quarry. Under major external triggers, both concretions and shales are equal in recording previous animal life (Fig. 7C), which can be clearly observed by the similar patterns of Ds, and Es, and consequently 3 s for the Herefordshire, Chengjiang, and Walcott Quarry Lagerstätten (Fig. 7A). Despite the observed similarities between the Walcott Quarry and the Chengjiang Biota, there are some differences in their pattern of preservation particularly in their conditional probabilities (Saleh et al., 2020). These differences might suggest subtle variations in the degree of biological involvement in shale preservation, as it is the case for concretions. However, more work is needed to test this hypothesis. Particularly, the role of mineralization (i.e., pyritization, silicification, and phosphatization) in efficiently replicating different biological structures (e.g., cuticle, epidermis, internal organs) while they decay on shale beds needs to be further investigated and constrained using geochemical approaches in addition to decay and mineralization experiments.

## Conclusion

The modes of soft tissue preservation of Mazon Creek, Herefordshire, and Montceau-les-Mines Konservat-Lagerstätten were investigated using a probability-based model. The obtained data show that even in sites that share the same mode of preservation, such as in the biologically mediated sideritic Konservat-Lagerstätten of Montceau-les-Mines and Mazon Creek, there are distinct variations in the probability of soft tissues being preserved. This is most likely due to differing environmental factors that influenced the speed of siderite formation in addition to the amount of decay that took place in the lead up to the precipitation of the concretion. In the Herefordshire Lagerstätte, a major volcanic event triggered concretion formation by supersaturating the ocean locally, there was little to no biological control, and fossil formation can be regarded as externally forced. When compared to shale preservation, the externally forced Herefordshire probabilities are very similar to sites such as the Chengjiang Biota and the Walcott Quarry. When major external events are lacking or delayed, concretionary sites such as Montceau-les-Mines and Mazon Creek better preserve internal organs and cellular sheets in contact with seawater than sites preserving fossils in shales such as the Fezouata Biota. This study shows that using probabilistic statistical methods to compare vastly different fossil deposits can provide unexpected insights into explaining preservational variation across Konservat-Lagerstätten.

### Supplementary Information

**Additional file 1.** Raw data file. 

## Data Availability

All the data needed to reproduce this manuscript are available in the main text and in the Additional file 1.
